# AIF and Scythe (Bat3) Regulate Phosphatidylserine Exposure and Macrophage Clearance of Cells Undergoing Fas (APO-1)-Mediated Apoptosis

**DOI:** 10.1371/journal.pone.0047328

**Published:** 2012-10-15

**Authors:** Giulio Preta, Bengt Fadeel

**Affiliations:** Division of Molecular Toxicology, Institute of Environmental Medicine, Karolinska Institutet, Stockholm, Sweden; Sudbury Regional Hospital, Canada

## Abstract

Phosphatidylserine (PS) exposure on the cell surface has been considered a characteristic feature of apoptosis and serves as a molecular cue for engulfment of dying cells by phagocytes. However, the mechanism of PS exposure is still not fully elucidated. Here we show that the cytosolic release from mitochondria of apoptosis-inducing factor (AIF) is required for PS exposure during death receptor-induced apoptosis and for efficient clearance of cell corpses by primary human macrophages. Fas-triggered PS exposure was significantly reduced upon siRNA-mediated silencing of AIF expression and by inhibition of the cytosolic translocation of AIF. In addition, AIF localizes to the plasma membrane upon Fas ligation and promotes activation of phospholipid scrambling activity. Finally, cytosolic stabilization of AIF through interaction with Scythe is shown to be involved in apoptotic PS exposure. Taken together, our results suggest an essential role for AIF and its binding partner Scythe in the pathway leading to apoptotic corpse clearance.

## Introduction

Apoptosis is a multistep process characterized by different morphological and biochemical alterations including cell shrinkage, membrane blebbing, nuclear condensation and DNA degradation [1]. These events are orchestrated by the activity of a family of cysteine proteases called caspases. Activation of caspases is initiated downstream of death receptor ligation in the plasma membrane (extrinsic apoptosis signaling) or upon release of pro-apoptotic factors such as cytochrome c from mitochondria (intrinsic apoptosis signaling). Death receptor triggering results in the formation of a Death-Inducing Signaling Complex (DISC) leading to activation of caspase-8, the most apical caspase in the extrinsic pathway, while cytochrome c promotes the formation of the apoptosome complex, resulting in capase-9 activation [2]. Both pathways converge on the activation of caspase-3, the central “executioner” of apoptosis. Mitochondrial outer membrane permeabilization also leads to the release of apoptosis-inducing factor (AIF) resulting in caspase-independent chromatin condensation and DNA fragmentation [3].

Ultimately, apoptotic cells are recognized and engulfed by neighboring phagocytic cells [4]. Indeed, clearance of apoptotic cell corpses defines the “meaning” of cell death as unengulfed cells may trigger unwanted inflammatory and/or autoimmune reactions [5]. Phosphatidylserine (PS) externalization on the surface of apoptotic cells was first described two decades ago [6] and we and others subsequently demonstrated this to be a critical step for macrophage engulfment of apoptotic cells [7], [8]. PS is recognized either directly by specific macrophage receptors for PS or indirectly via PS-binding bridging proteins such as MFG-E8 [9]. Of note, MFG-E8-deficient mice display systemic autoimmune disease, thus underscoring the importance of PS-mediated cell clearance in vivo [10]. Whereas significant advances have been made in recent years to elucidate the molecular mechanisms involved in the execution of cell death, the removal of apoptotic cells by macrophages has received less attention [9]. In particular, the factors influencing the expression of “eat-me” signals such as PS on the surface of apoptotic cells remain to be elucidated. Lipid scrambling in the plasma membrane with surface exposure of PS is believed to be a critical step in apoptosis but the specific protein responsible of the scrambling activity as well as factors promoting scramblase activation are still not fully identified [11]. Suzuki et al. [12] recently reported on the identification of a protein that is essential for Ca^2+^-dependent phospholipid scrambling, but it remains unproven whether the same pathway is engaged in cells undergoing apoptosis. In addition, it has been shown that the worm AIF homologue (WAH-1) in *Caenorhabditis elegans* promotes PS externalization in apoptotic cells through association with phospholipid scramblase 1 (SCRM-1) and subsequent activation of its phospholipid scrambling activity [13]. This is suggestive of a role for AIF in PS exposure during apoptosis. Indeed, Susin et al. reported more than one decade ago that microinjected AIF induces collapse of the mitochondrial transmembrane potential (ΔΨ_m_) and exposure of PS in mammalian cells [3]. Moreover, we previously reported that overexpression of Bcl-2 prevents PS exposure following Fas ligation in so-called type I cells while DNA fragmentation remained unaffected [14] thus underscoring the role of mitochondria and/or mitochondrial factor(s) for PS exposure. Finally, we recently demonstrated that re-localization of Scythe (Bat3) from the nucleus to the cytosol is required for PS exposure [15]. The mechanism linking Scythe to PS exposure, however, was not determined.

Here, we investigated the role of AIF in macrophage clearance of apoptotic cells following ligation of the Fas death receptor or treatment with the protein kinase inhibitor, staurosporine, a universal inducer of (mitochondria-dependent) apoptosis. We show that the down-regulation of AIF expression by specific siRNA results in a reduction of the scramblase activity in apoptotic cells and a concomitant decrease of PS exposure and subsequent phagocytosis by primary human macrophages. Moreover, we report the formation of a complex between AIF and Scythe, a protein that was previously shown to stabilize AIF in the cytosol in cells subjected to ER stress [16], and show that this complex is critical for PS exposure and phagocytosis of apoptotic cells by macrophages. These findings shed new light on the molecular players in the process of PS exposure during apoptosis and may yield novel targets for drugs to control chronic inflammation and autoimmune disease.

## Results

### Down-regulation of AIF Reduces Fas-triggered PS Exposure in Jurkat Cells

**Figure 1:**
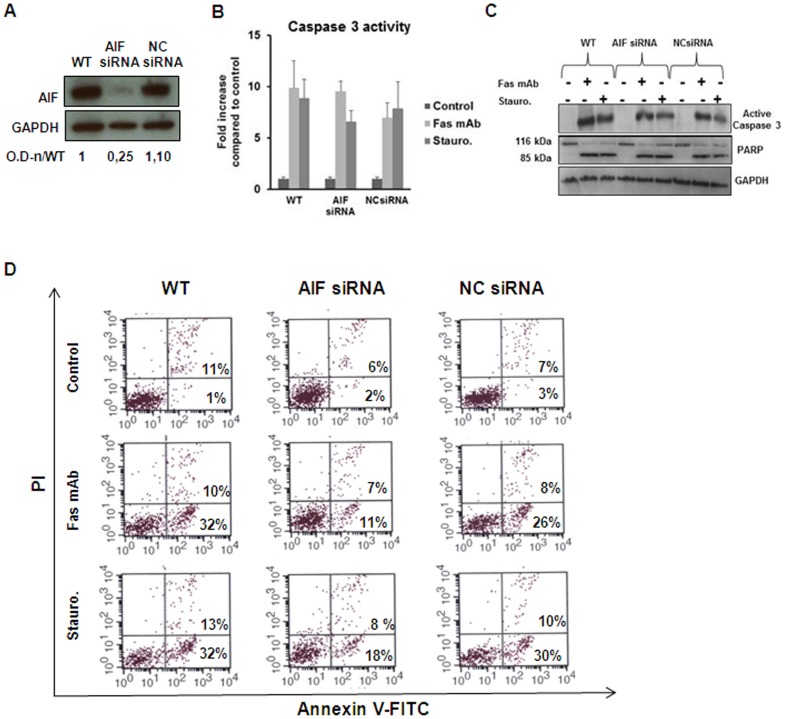
Down-regulation of AIF reduces PS exposure in Jurkat cells without affecting caspases activation. A. Jurkat cells were transfected with siRNA for AIF and non-targeting siRNA (NC) in 12-well culture plates. After 48 h cells were collected, lysed in RIPA buffer and Western blotting analysis was performed to detect the levels of AIF. GAPDH was used as loading control. Quantification was made using the program Image J and the values of optical density (OD) were normalized according to the levels of GAPDH. B. Jurkat cells transfected like in A were treated with Fas mAb (50 ng/ml) or staurosporine (0.5 µM) and after 3 h were lysed and enzymatic assay for caspase-3 was performed using the fluorogenic substrate DEVD-AMC. Results are reported as fold increase compared to control. Data are presented as the mean± S.D of nine values derived from three different experiments performed in triplicate (one-way ANOVA *Dunnett’s* test). C. Jurkat cells transfected like in A were treated with Fas mAb (50 ng/ml) or staurosporine (0.5 µM). After 3 h, cells were collected and lysed and Western blotting was performed to detect active caspase-3 and PARP cleavage. GAPDH was used as loading control. D. Jurkat cells transfected like in A were treated with Fas mAb (50 ng/ml) or staurosporine (0.5 µM). After 3 h, cells were collected and Annexin V-FITC staining was performed to determine the level of PS exposure.

To investigate the role of AIF in apoptotic PS exposure we performed knockdown experiments using siRNA against AIF (Fig. 1A and Supplementary Fig. 1A). Caspase-3 activation measured by enzymatic assay, using the fluorogenic substrate DEVD-AMC (Fig. 1B), as well as the levels of expression of active caspase-3 and PARP cleavage (a marker of caspase-3 activation) (Fig. 1C and Supplementary Fig. 1B) were not influenced by the decreased levels of AIF protein following treatment of Jurkat cells with agonistic Fas mAb (50 ng/ml) and staurosporine (0.5 µM). Annexin V labeling, however, revealed a decrease in PS exposure in AIF knockdown Jurkat cells compared to untreated cells or cells treated with non-targeting siRNA (Fig. 1D and Supplementary Fig. 1C). These results strongly suggest that AIF, a key mediator of caspase-independent apoptosis-like programmed cell death [17], plays an active role in the pathway leading to PS exposure, thus confirming the role of mitochondria as critical organelles for signals regulating the phagocytosis process as suggested in previous work [14], [18].

### Inhibition of AIF Cytosolic Release Reduces PS Exposure in Fas-treated Jurkat Cells

**Figure 2:**
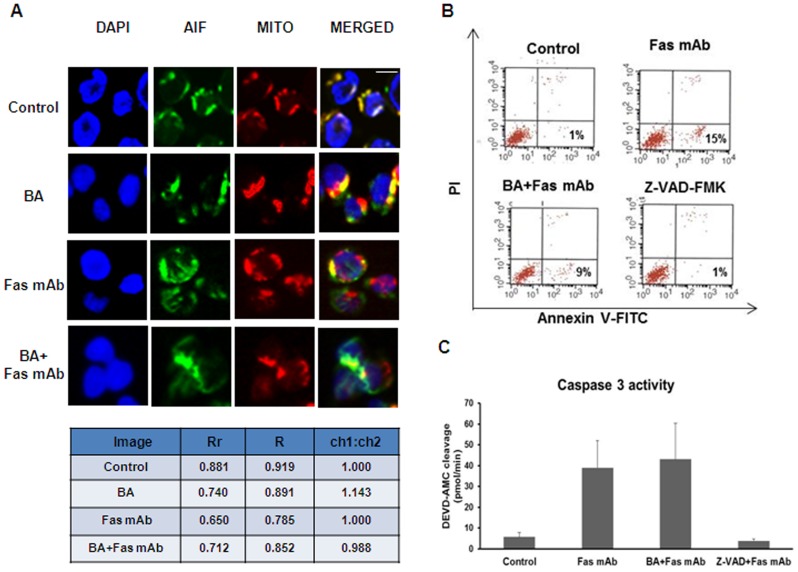
Inhibition of AIF cytosolic release reduces PS exposure in Fas-treated Jurkat cells. A. Jurkat cells were treated with Fas mAb (50 ng/ml) with or without pre-treatment for 2 h with bongkrekic acid (BA) (50 µg/ml). Cells were stained with the mitochondrial marker, MitoTracker Red. Co-localization of AIF and mitochondria was validated by image analysis using the program Image J. B. Jurkat cells treated as above were collected after 3 h and Annexin V-FITC staining was performed to monitor the level of PS exposure. C. Jurkat cells treated as above were lysed and real-time in vitro assay for caspase-3 was performed using the fluorogenic substrate DEVD-AMC. Results are reported as pmol/min of substrate cleavage. Data are presented as the mean± S.D of nine values derived from three different experiments performed in triplicate (one-way ANOVA *Dunnett’s* test).

To further assess the importance of AIF we used a different approach, whereby the translocation of AIF from mitochondria to cytosol upon apoptosis induction was blocked. Fas ligation is known to induce extensively the re-localization of AIF from mitochondria to cytosol [19]. Bongkrekic acid (BA) is a specific ligand of the mitochondrial adenine nucleotide translocator and acts as an efficient inhibitor of mitochondrial permeability transition pore (PTP) opening and AIF release from mitochondria [20]. Jurkat cells were incubated with Fas mAb (50 ng/ml) in the presence or not of BA (50 µg/ml). Immunocytochemistry analysis showed, as expected, an inhibition of cytosolic release of AIF in BA+Fas mAb-treated Jurkat cells and an increased degree of co-localization of AIF with the mitochondrial marker MitoTracker-Red compared with Jurkat cells treated with Fas mAb alone (Fig. 2A, co-localization validated by Image J analysis software). Annexin V staining showed a decrease in the rate of PS exposure in BA+Fas mAb-treated Jurkat cells compared to Jurkat treated with Fas mAb alone (Fig. 2B). However, the levels of caspase-3 activation were unchanged (Fig. 2C) confirming that the inhibition of PTP opening and subsequent AIF release affects PS exposure in this model without influencing caspase activation.

### AIF Localizes to Lipid Rafts and is Required for the Activation of Phospholipid Scramblase

**Figure 3:**
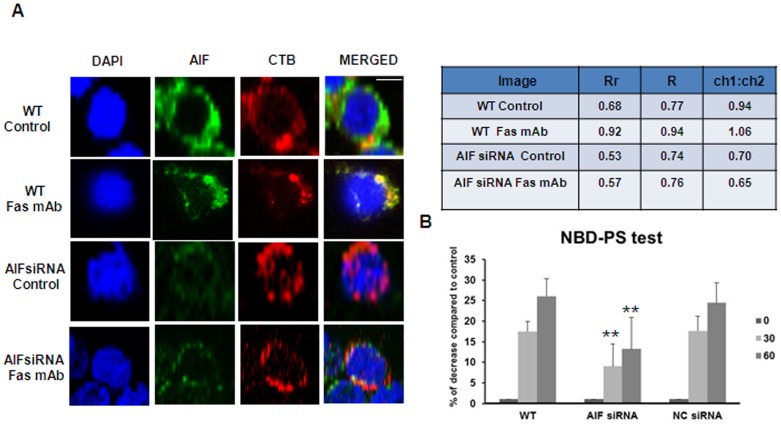
AIF is required for the activation of the phospholipid scramblase activity. A. Jurkat cells WT and knockdown for AIF (48 h) were stained with the lipid raft marker, cholera toxin B (CTB) and with an anti-AIF antibody and subjected to immunofluorescence imaging. Co-localization between AIF and lipid rafts was validated by image analysis using the program Image J. B. Fluorescent NBD-PS was used to monitor scramblase activity in apoptotic cells. Following transfection of Jurkat cells with siRNA as indicated (48 h), cells were incubated with NBD-PS (10 µM) for 10 min at 28°C. Then, cells were treated with 50 ng/ml Fas mAb for 3 h, after which the percentage of residual fluorescence (due to the reduction of the NBD-PS probe) was determined using a Fluoroscan II plate reader with an excitation wavelength of 470 nm and emission wavelength = 540 nm. Data are presented as the mean± S.D of six values derived from two different experiments performed in triplicate. *P<0.05 (one-way ANOVA *Dunnett’s* test).

PS exposure during apoptosis involves the coordinated inhibition of aminophospholipid translocase activity and activation of a phospholipid scramblase activity resulting in the collapse of phospholipid asymmetry in the plasma membrane [21]. The precise molecular identity of the phospholipid scramblase in mammalian cells has not been deduced; however, previous reports have shown that phospholipid scramblase 1 (PLSCR1) is enriched in lipid rafts [22], [23]. We thus hypothesized that AIF could activate phospholipid scramblase activity through its association with lipid rafts. To this end, we performed immunofluorescence analysis using cholera toxin B (CTB), a lipid raft marker, in WT and AIF siRNA-transfected Jurkat cells. AIF displayed co-localization with CTB after Fas mAb treatment in WT cells; knockdown of AIF does not seem to influence the pattern of lipid raft expression (Fig. 3A, co-localization validated by Image J analysis software). To verify if AIF plays any role in the promotion of scramblase activation in Fas-triggered Jurkat cells we assessed scramblase activity by measuring transbilayer movement of NBD-labeled phospholipid analogues, as previously described [24]. To this end, Jurkat cells transfected or not with siRNA against AIF or with non-targeting siRNA were allowed to internalize NBD-PS, and were then subjected to anti-Fas mAb treatment (50 ng/ml) for 3 h, followed by real-time measurement of fluorescence using a spectrofluorimeter. Non-internalized NBD-PS was extracted using bovine serum albumin prior to the measurement of cellular fluorescence. In WT Jurkat cells and in cells transfected with control siRNA, Fas triggering resulted in a time-dependent decrease in cellular fluorescence, indicative of outward movement of internalized NBD-PS (Fig. 3B). Scramblase activation was, however, significantly reduced in Jurkat cells transfected with siRNA against AIF (efficiency of AIF knockdown reported in Fig. 3A). Taken together, these findings suggest a role – direct or indirect – for AIF in scramblase activation, consistent with the role of WAH-1 in activation of SCRM-1 in *C. elegans* [13].

### AIF and Scythe are Required for Macrophage Clearance of Fas-triggered Jurkat Cells

**Figure 4:**
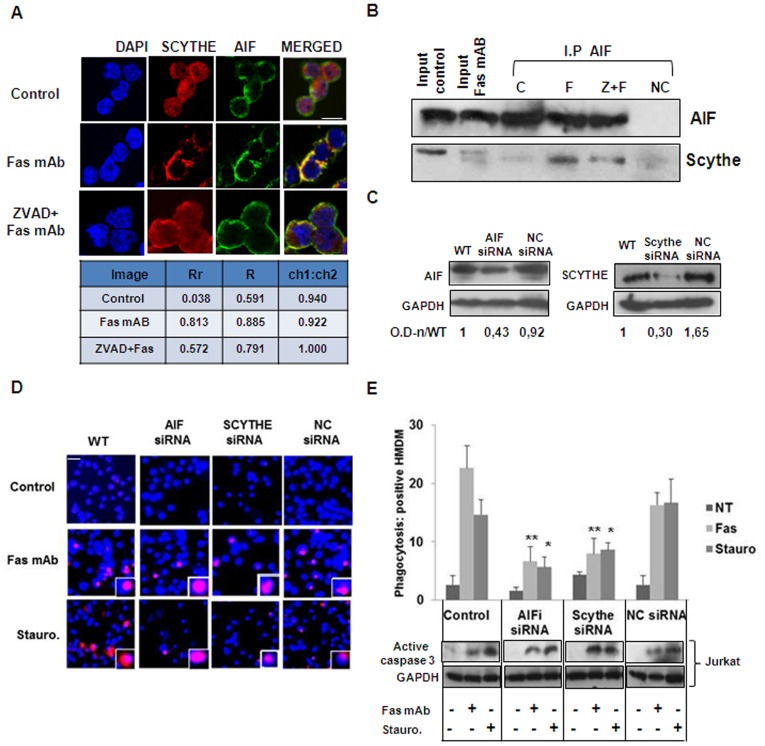
Cytosolic interaction between AIF and Scythe is required for macrophage clearance of Fas-triggered Jurkat cells. A. Immunocytochemistry analysis of Scythe and AIF in Jurkat cells un-treated and treated for 3 h with Fas mAb (250 ng/ml) in the presence or absence of the pan-caspase inhibitor, Z-VAD-FMK (20 µM). Nuclei are stained in blue (DAPI). B. Jurkat cells treated like in A were lysed and AIF was then immunoprecipitated. Western blot is 10% of the input and is derived from both control and Fas mAb treated cells. Negative control (NC) consists of glass beads incubated with normal rabbit serum. C. Jurkat cells were transfected with siRNA for AIF, Scythe and non-targeting siRNA (NC). After 48 h cells were collected, lysed in RIPA buffer and Western blotting analysis was performed to detect the levels of AIF. GAPDH was used as loading control. Quantification was made using the program Image J and the values of optical density (OD) were normalized to the levels of GAPDH. D. Jurkat cells, treated with siRNA for AIF, Scythe or NC were induced to undergo apoptosis by Fas mAb (50 ng/ml) or staurosporine (0.5 µM). After 3 h cells were co-cultivated with human monocyte-derived macrophages (HMDM) for 1 h. Examples of phagocytosis of TAMRA-labelled Jurkat cells (red) are depicted. Nuclei of macrophages and engulfed target cells are stained with DAPI (blue). E. Phagocytosis is reported as the percentages of macrophages positive for uptake of target cells. Data are presented as the mean ± S.D of six values derived from two different experiments performed in triplicate. *P<0.05; **P<0.01 (one-way ANOVA *Dunnett’s* test). Caspase-3 activation in WT versus AIF or Scythe siRNA-transfected cells determined by immunoblotting is shown in the panel below. GAPDH is used as loading control.

Scythe localization and redistribution in apoptotic cells is highly debated. According to one study [25], Scythe is a nuclear protein that contains an active C-terminal nuclear localization sequence (NLS) and induction of apoptosis by staurosporine does not cause redistribution or cleavage of Scythe, suggesting that this protein remains localized in the nucleus during apoptosis and does not interact with elements of the apoptotic machinery in the cytosol. In contrast, a recent report has shown that the majority of endogenous Scythe can be found in the cytosolic fraction in multiple mouse primary tissues [26]. Others have reported that Scythe re-localizes to the cytosol after thapsigargin treatment [16] or after ricin treatment following caspase-3 cleavage [27]. We previously observed that Scythe is cleaved by caspase-3 during Fas mAb- and staurosporine-induced apoptosis and we noted that the larger fragment re-localizes to the cytosol [15]. To investigate in detail the re-localization and possible molecular interactions of Scythe following apoptosis induction, we treated Jurkat cells with Fas mAb using a dose (250 ng/ml) which guarantees an extensive cleavage and cytosolic translocation of Scythe as previously demonstrated [15]. Immunofluorescence analysis showed that Scythe, located in the nucleus in control cells, re-localizes to the cytosol after Fas mAb treatment, and this re-localization is blocked by pre-treatment with the pan-caspase inhibitor, Z-VAD-FMK in line with previous work [15]. Moreover, co-localization of Scythe and AIF was observed and validated using image analysis software (Fig. 4A). Next, we demonstrated an interaction between AIF and Scythe by immunoprecipitation. Jurkat cells were left untreated or treated with Fas mAb in the presence or not of Z-VAD-FMK and AIF was then immunoprecipitated using a rabbit anti-AIF antibody. Western blotting analysis revealed the cleaved form of Scythe as binding partner of AIF following apoptosis induction (Fig. 4B and full blots in Supplementary Fig. 2). To evaluate the importance of Scythe interaction for the cytosolic stabilization of AIF and for subsequent PS exposure and phagocytosis by macrophages, we performed specific siRNA against AIF or Scythe (Fig. 4C). TAMRA-labeled Jurkat cells transfected or not with siRNA against AIF, Scythe, or control siRNA were induced to undergo apoptosis by Fas mAb (50 ng/ml) or staurosporine (0.5 µM) treatment and were then co-cultured with human monocyte-derived macrophages (HMDM). As seen in Fig. 4D–E, phagocytosis of apoptotic Jurkat cells was clearly decreased when the expression of AIF or Scythe was reduced, thus confirming the importance of these proteins for cell clearance of apoptotic cells. However, the degree of caspase-3 activation was unchanged in Jurkat cells in which Scythe or AIF expression was silenced (Fig. 4E), in line with the results shown in Fig. 1C. In other words, the reduction of phagocytosis is not merely due to a reduced level of apoptosis but seems to correlate with the level of PS exposure.

## Discussion

This study demonstrates the importance of AIF and its binding partner Scythe for the externalization of PS on the surface of apoptotic cells and the subsequent elimination of these cells by primary human macrophages. This work further underscores the importance of mitochondria-to-plasma membrane signaling in PS exposure during apoptosis, in line with previous studies in the worm [13]. Hence, while cytochrome c-dependent signaling for the execution of apoptosis is not recapitulated in the worm [2], AIF (WAH-1) signaling for apoptotic cell clearance is strongly conserved [13], although our findings suggest that in mammalian cells the process leading to programmed cell clearance is more multifaceted, as AIF requires the contribution of another protein (Scythe) for its cytosolic stabilization. Scythe, in turn, has previously been implicated in apoptosis signaling in *Drosophila melanogaster* [28], [29].

We and others have shown that PS exposure and phagocytic clearance of apoptotic cells can be dissociated from apoptosis induction and attendant nuclear changes, such as DNA fragmentation [14], [18]. Disruption of mitochondrial function during apoptosis was shown to occur through caspase cleavage of the p75 subunit of complex I of the electron transport chain [30]. Interestingly, in cells expressing a non-cleavable p75 mutant, a delay in PS externalization was observed while cytochrome c release and DNA fragmentation were unaffected. Furthermore, van Delft et al. [31] reported that caspase-9-deficient cells, while initially refractory to apoptotic insults, eventually succumb to caspase-independent cell death with exposure of PS and recognition and engulfment by phagocytes. The latter study showed that the exposure of PS coincided with the collapse of the mitochondrial transmembrane potential, suggesting a role for mitochondria and/or mitochondrial factor(s), but the mechanism of PS exposure was not determined. Our current data provide a potential explanation for these observations namely that in cells undergoing apoptosis, AIF is released from mitochondria to the cytosol, where it is stabilized by Scythe resulting in activation of phospholipid scramblase at the plasma membrane and PS exposure on the cell surface, leading to clearance by macrophages.

According to our model, the redistribution of Scythe from the nucleus to the cytosol is a necessary step in apoptotic PS exposure, presumably in order to stabilize AIF in the cytosol, as shown here and in a previous study of thapsigargin-treated cell [16]. The importance of Scythe for PS exposure is suggested by previous studies using Scythe-deficient murine embryonic fibroblasts (MEF) which displayed a decrease in Annexin V-positive cells following different apoptotic stimuli, including thapsigargin, Fas mAb and staurosporine [16]. Moreover, we recently reported that the silencing of Scythe using specific siRNA Scythe prevented PS exposure in Jurkat cells [15]. Overall, our findings underscore the importance of redistribution of apoptotic factors, including translocation of AIF from mitochondria to the cytosol, and of Scythe from the nucleus to the cytosol, in the regulation of PS exposure at the plasma membrane. Our data also provide a tentative explanation for the numerous previous observations of caspase-dependent PS exposure [32], [33]. However, according to our results, caspase activation is a required but not sufficient step to promote apoptotic PS exposure as Scythe cleavage and its re-localization from the nucleus to the cytosol is a caspase-dependent event [15], whereas blocking of AIF release from mitochondria to the cytosol leads to a decrease in PS exposure even in presence of caspase activity. Norberg et al. have reported that silencing of AIF expression in HEK293 cells leads to a reduction of annexin V-positive cells following staurosporine treatment [34], providing further support for our model.

We previously demonstrated that the externalization of PS is necessary for efficient phagocytosis of Fas-triggered cells; the oxidation of PS further promoted cell clearance [8]. The present data support this conclusion and provide conclusive evidence that AIF is required for PS-dependent clearance of apoptotic cells. Others have recently reported that lymphoma cells transformed with a constitutively active form of TMEM16F, a putative phospholipid scramblase, exposed high levels of PS but failed to undergo phagocytosis [12], [35]. This intriguing finding suggests that PS exposure alone is not a sufficient signal for programmed cell clearance. However, it is remains possible (even likely) that the level of expression of PS needs to exceed a certain threshold before cells become substrates for macrophage engulfment [36] and/or that PS oxidation is also needed in order to promote efficient phagocytosis [8], [37].

Loss of plasma membrane phospholipid asymmetry and exposure of PS on the cell surface is a general feature of apoptosis [38], necessary for the generation of signals which provoke recognition of apoptotic cells by phagocytes [4]. Bratton et al. suggested that PS exposure results from the concurrent inhibition of aminophospholipid translocase activity and activation of a phospholipid scramblase activity [21]. However, the protein responsible for the scramblase activity in apoptotic cells as well as factors promoting scramblase activation is still not fully elucidated. Nonetheless, the current data, combined with previous work in the nematode [13], point to AIF as a key protein for scramblase activation during apoptosis. Moreover, our data suggest that this activation may occur in lipid rafts, which are known to play an essential role as platforms for signal transduction [39]. Experiments to prove a role of lipid rafts specifically for apoptotic PS exposure are difficult to perform, however, since Fas signaling also depends on the integrity of lipid rafts [40].

In conclusion, the present results point towards a critical and conserved role of mitochondria, and specifically for AIF, for the signaling events leading to phagocytic clearance of apoptotic cells i.e. programmed cell clearance [9]. The significance of this finding is underscored by the importance of effective disposal of apoptotic cells in normal development [41]. The swift and silent removal of apoptotic cells is also required to promote the resolution of inflammation and to prevent the inadvertent induction of autoimmune responses [42]. Deciphering the mechanism of PS-dependent clearance of apoptotic cells may therefore yield novel targets for the therapeutic control of chronic inflammation and autoimmune diseases.

## Materials and Methods

### Reagents and Cell Lines

Agonistic anti-Fas monoclonal antibodies (clone CH-11) were purchased from Medical & Biological Laboratories, Ltd (Nagoya, Japan). Staurosporine, bongkrekic acid, aspartate-glutamate-valine-aspartate-7-amino-4-methyl-coumarin (DEVD-AMC), and N-Benzyloxycarbonyl-Val-Ala-Asp(O-Me) fluoromethyl ketone (Z-VAD-FMK) were all from Sigma (St Louis, MO, USA). Recombinant caspase-3 and MitoTracker®Red were purchased from Invitrogen (San Diego, CA, USA), and NBD-PS was from Avanti Polar Lipids (Alabaster, AL, USA). Alexa 555-conjugated cholera toxin B was purchased from Molecular Probes (Eugene, Oregon, USA). The human T cell leukemic cell line, Jurkat were obtained from the European Collection of Cell Cultures (Salisbury, UK) and was cultured in RPMI-1640 medium (Sigma) supplemented with 10% heat-inactivated fetal bovine serum, 2 mM glutamine, 100 U/ml penicillin and 100 mg/ml streptomycin (Gibco Paisley, Scotland).

### RNA Interference

ON-TARGETplus Human Scythe (BAT3) siRNA, SiGENOME SMARTpool Human AIF siRNA, and ON-TARGETplus Non-targeting Pool (NC siRNA) were all purchased from Dharmacon (Lafayette, CO, USA). Jurkat cells were transfected for 48 h with 200 nM siRNA against AIF, Scythe or NC in complete growth medium using the *TransIT*-*TKO*® *Transfection* Reagent from Mirusbio (Madison, WI, USA) according to the manufacturers’ instructions.

### Caspase-3-like Activity

The measurement of DEVD-AMC cleavage was performed in a fluorometric assay as described previously [43]. The cleavage of the fluorogenic peptide substrate was monitored by AMC liberation in a TECAN Infinite® 200 plate reader (Labsystems, Stockholm, Sweden) using 355 nm excitation and 460 nm emission wavelengths. Fluorescence units were converted to pmol of AMC cleavage per minute based on a standard curve of free AMC.

### Phosphatidylserine Exposure

PS exposure was quantified by propidium iodide (PI) (Sigma) and Annexin V (Annexin-V-Fluos staining kit, Roche Diagnostics GmbH, Manheim, Germany) dual labeling as described previously [44]. Cells were analyzed by flow cytometry within 30 min after staining on a FACS Calibur (BD Biosciences) operating with CellQuest software (BD Biosciences). AV^+^PI^−^ and AV^+^PI^+^ cells were defined as early and late apoptotic cells, respectively.

### Western Blotting

For protein detection, western blotting was performed according to standard procedures. The following primary antibodies were used: GAPDH (Ambion Austin, TX, USA), goat and rabbit anti-AIF (Santa Cruz Biotechnology, Santa Cruz, CA, USA), mouse anti-Scythe (Abcam, Cambridge, United Kingdom). cleaved caspase-3 (AH Diagnostics, Stockholm, Sweden) and PARP (BIOMOL, Plymouth Meeting, PA, USA). After washing, the membranes were incubated with a peroxidase-conjugated secondary antibody (Pierce, Rockford, IL, USA) and bound antibody was visualized by enhanced chemiluminescence (Pierce).

### Immunoprecipitation

For immunoprecipitation, rabbit anti-AIF (Santa Cruz Biotechnology) was added to the cell lysates at 4°C overnight, and then protein G Sepharose (GE Healthcare, *Piscataway*, NJ, USA) was added for 4 h. After centrifugation, glass beads were removed by heating and precipitated immune complexes were re-suspended in SDS sample buffer for Western blotting analysis.

### Immunocytochemistry

To analyze cellular localization of AIF and Scythe, cells were treated for 3 h with Fas mAb in the presence or absence of the pan-caspase inhibitor Z-VAD-FMK (1 h pre-incubation) or bongkrekic acid (2 h pre-incubation). After incubation at 37°C, cells were attached to glass slides through cytospin and fixed in PFA 3.7% for 30 min; then, the slides were rehydrated in PBS for 1 h. The goat anti-AIF and rabbit anti-Scythe antibodies (300 sc98519 from Santa Cruz Biotechnology) were added and remained overnight at 4°C until a brief wash in PBS, after which a secondary conjugate (Alexa or FITC-conjugated) was added and incubated for 1 h at RT. Then, the slides were washed and stained with Vecta shield containing DAPI (Vector Laboratories Inc. Burlingame, CA, USA) and analyzed by an inverted Nikon ECLIPSE TE2000-S fluorescence microscope (Nikon Corporation, Kanagawa, Japan) operating with NIS-Elements software (Nikon). The co-localization analysis was performed with Image J software using high resolution confocal images acquired from a Leica microscope (Leica Microsystems, Wetzlar, Germany). Rr is the Pearson’s correlation coefficient, which range from 1 to −1. A value of 1 represents perfect correlation, −1 represents perfect exclusion and zero represents random localization. R is Mander’s Overlap coefficient, which ranges between 1 and zero with 1 being high-co-localization, zero being low. Ch1:Ch2 represents instead the red: green pixel ratio. The correlation coefficients are derived from a field containing at least 10 cells.

### Scramblase Activity

Fluorescent NBD-PS was used to monitor scramblase activity in apoptotic cells as previously described [24]. Unincorporated PS was back-extracted with 1% BSA. This back-extraction has been shown to remove NBD-labeled lipid from the surface of the cell membrane, leaving only that which has flipped to the interior leaflet of the membrane. Triton X-100 (0,3% final concentration) was added to make all NBD probe available to BSA thus allowing determination of the background fluorescence. Then cells were treated with 50 ng/ml Fas mAb for 3 h, after which the percentage of residual fluorescence (due to the reduction of the NBD-PS probe) was determined using a Fluoroscan II plate reader (Thermo Electron Co) operating with the following instrumental conditions: excitation wavelength = 470 nm, emission wavelength = 540 nm.

### Phagocytosis Assay

Peripheral blood mononuclear cells (PBMC) were prepared from buffy coats obtained from anonymized blood donors (Karolinska University Hospital, Stockholm, Sweden) by density gradient centrifugation using Lymphoprep (Axis-Shield, Dundee, UK) and were positively selected for CD14 expression (Miltenyi Biotec, Bergisch-Gladbach, Germany) to obtain HMDM as previously described [8]. To determine the degree of engulfment of apoptotic cells, Jurkat cells were pre-stained with tetramethylrhodamine (TAMRA) (Sigma) and then treated with Fas mAb and staurosporine for 3 h. Jurkat cells were then added to M-CSF-activated HMDM at a ratio of 5∶1. HMDM and target cells were co-cultured at 37°C for 1 h. After co-cultivation, non-ingested target cells were washed off with several washes in cold PBS, and the remaining cells were fixed in 2% paraformaldehyde for 15 min and then stained with Hoechst 33342 (1 µg/ml) (Sigma). Phagocytosis was evaluated by counting macrophages in visual light and thereafter, counting macrophage-engulfed, TAMRA-labeled cells under UV illumination using an inverted Nikon ECLIPSE TE2000-S fluorescence microscope (Nikon) equipped with a DS-5M digital camera operating with NIS-Elements software (Nikon). A minimum of 300 macrophages per experimental condition was analyzed. Data are reported as the percentage of HMDM positive for uptake of TAMRA-labeled target cells.

### Statistics

*Data* are *expressed as mean* ± S.D. Statistical comparisons between groups were performed using analysis of variance (one way-ANOVA *Dunnett’s* test) with Graphpad Software. Differences between samples were considered to be significant at P<0.05.

## Supporting Information

Figure S1 **(A) Western blot showing knockdown of AIF: samples correspond to** **Figure 1B** **, in which data from n = 3 experiments are reported (AIF knockdown in the third sample is shown in** **Figure 1A** **).** Jurkat cells were transfected with siRNA for AIF and non-targeting siRNA (NC). After 48 h cells were collected, lysed in RIPA buffer and Western blotting analysis was performed to detect the levels of AIF. GAPDH was used as loading control. Quantification was made using the program Image J and the values of optical density (OD) were normalized according to the levels of GAPDH. **(B)** PARP cleavage and caspase-3 activation in samples corresponding to Figure 1B (for a total of n = 3; PARP cleavage data from the third sample are shown in Figure 1C). Jurkat cells transfected as indicated above were treated with Fas mAb (50 ng/ml) or staurosporine (0.5 µM). After 3 h, cells were collected and lysed and Western blotting was performed to detect active caspase-3 and PARP cleavage. GAPDH was used as loading control. **(C)** Jurkat cells transfected as indicated in panel A were treated with Fas mAb (50 ng/ml) or staurosporine (0.5 µM). After 3 h, cells were collected and Annexin V-FITC staining was performed to determine the percentage PS exposure. Note that the samples are the same as those shown in Figure 1D. In the latter figure, the percentages of cells with exposed PS are reported while here the levels of PS exposed per cell are indicated (as the data are plotted on a Log scale the X Geometric mean values are indicated).
(TIF)

Figure S2 **Full-size blots of the immunoprecipitation experiment shown in** **Figure 4B** **. Jurkat cells treated with Fas mAb (250 ng/ml) for 3 h, in the presence or not of Z-VAD-FMK (20 µM) were lysed and AIF was then immunoprecipitated.** Western blot is 10% of the input and is from both control and Fas mAb treated cells. Negative control (NC) consists of glass beads incubated with normal rabbit serum. **(A)** anti-AIF and **(B)** anti-Scythe immunoblotting. The immunoprecipitated Scythe corresponds to the cleaved form since it has the molecular weight of the lower band present in the Fas mAb-treated input.
(TIF)
