# Supplementary figures and images for: AIF and Scythe (Bat3) Regulate Phosphatidylserine Exposure and Macrophage Clearance of Cells Undergoing Fas (APO-1)-Mediated Apoptosis
Source: PLoS One. 2012 Oct 15;7(10):e47328. doi: 10.1371/journal.pone.0047328 (PMC3471829; doi:10.1371/journal.pone.0047328)

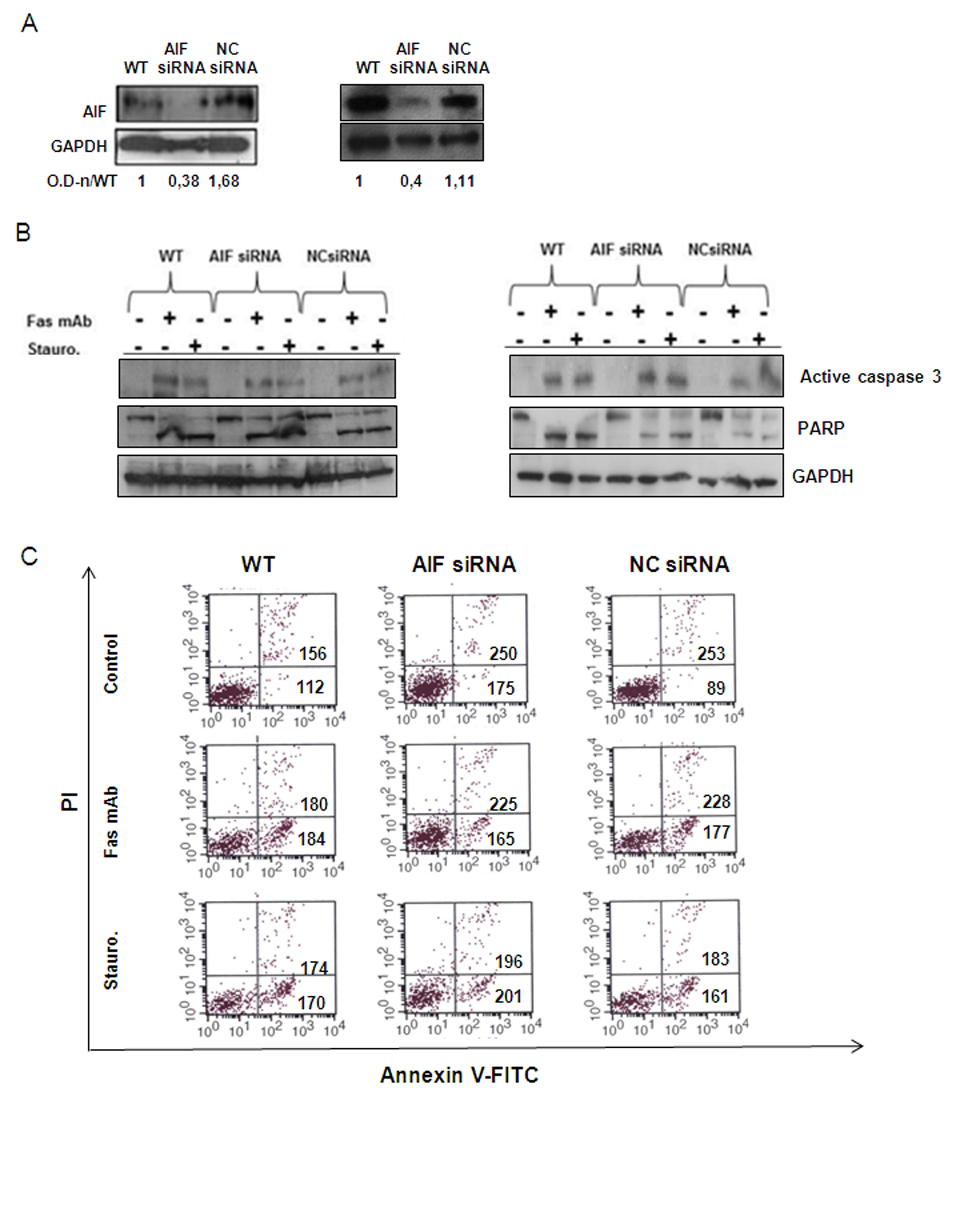

Supplement: Figure S1 — (A) Western blot showing knockdown of AIF: samples correspond to Figure 1B , in which data from n = 3 experiments are reported (AIF knockdown in the third sample is shown in Figure 1A ). Jurkat cells were transfected with siRNA for AIF and non-targeting siRNA (NC). After 48 h cells were collected, lysed in RIPA buffer and Western blotting analysis was performed to detect the levels of AIF. GAPDH was used as loading control. Quantification was made using the program Image J and the values of optical density (OD) were normalized according to the levels of GAPDH. (B) PARP cleavage and caspase-3 activation in samples corresponding to Figure 1B (for a total of n = 3; PARP cleavage data from the third sample are shown in Figure 1C). Jurkat cells transfected as indicated above were treated with Fas mAb (50 ng/ml) or staurosporine (0.5 µM). After 3 h, cells were collected and lysed and Western blotting was performed to detect active caspase-3 and PARP cleavage. GAPDH was used as loading control. (C) Jurkat cells transfected as indicated in panel A were treated with Fas mAb (50 ng/ml) or staurosporine (0.5 µM). After 3 h, cells were collected and Annexin V-FITC staining was performed to determine the percentage PS exposure. Note that the samples are the same as those shown in Figure 1D. In the latter figure, the percentages of cells with exposed PS are reported while here the levels of PS exposed per cell are indicated (as the data are plotted on a Log scale the X Geometric mean values are indicated). (TIF) [file pone.0047328.s001.tif]

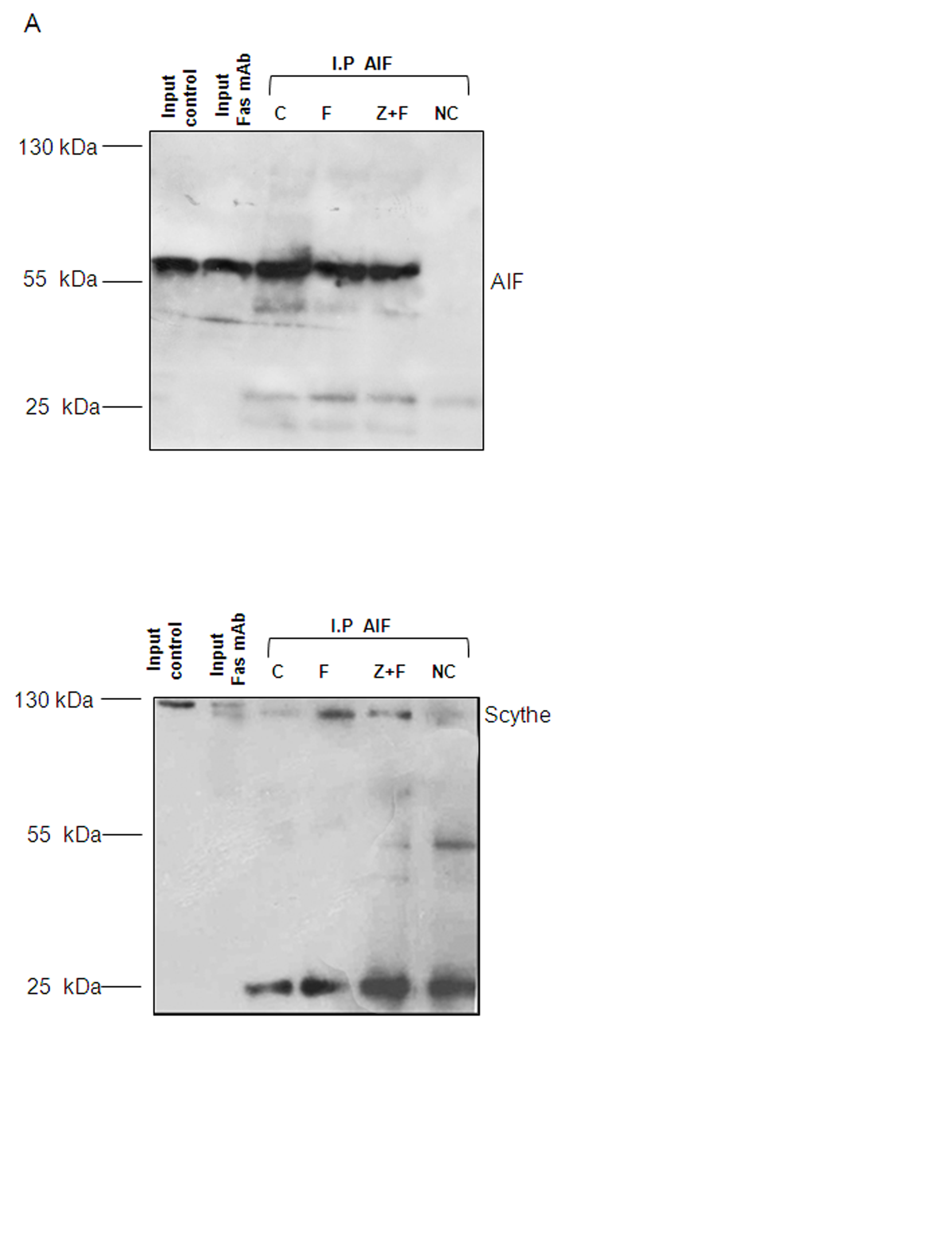

Supplement: Figure S2 — Full-size blots of the immunoprecipitation experiment shown in Figure 4B . Jurkat cells treated with Fas mAb (250 ng/ml) for 3 h, in the presence or not of Z-VAD-FMK (20 µM) were lysed and AIF was then immunoprecipitated. Western blot is 10% of the input and is from both control and Fas mAb treated cells. Negative control (NC) consists of glass beads incubated with normal rabbit serum. (A) anti-AIF and (B) anti-Scythe immunoblotting. The immunoprecipitated Scythe corresponds to the cleaved form since it has the molecular weight of the lower band present in the Fas mAb-treated input. (TIF) [file pone.0047328.s002.tif]
